# Assessing Carnivorous Plants for the Production of Recombinant Proteins

**DOI:** 10.3389/fpls.2019.00793

**Published:** 2019-06-19

**Authors:** Sissi Miguel, Estelle Nisse, Flore Biteau, Sandy Rottloff, Benoit Mignard, Eric Gontier, Alain Hehn, Frédéric Bourgaud

**Affiliations:** ^1^Plant Advanced Technologies SA, Vandoeuvre-lès-Nancy, France; ^2^Laboratoire Agronomie et Environnement, INRA, Université de Lorraine, Vandoeuvre-lès-Nancy, France; ^3^Laboratoire Biopi, Université de Picardie Jules Verne, Amiens, France

**Keywords:** carnivorous plant, recombinant protein, secretion-based platform Drosera, *Nepenthes*, agroinfiltration, virus-based plant transformation, protease

## Abstract

The recovery of recombinant proteins from plant tissues is an expensive and time-consuming process involving plant harvesting, tissue extraction, and subsequent protein purification. The downstream process costs can represent up to 80% of the total cost of production. Secretion-based systems of carnivorous plants might help circumvent this problem. *Drosera* and *Nepenthes* can produce and excrete out of their tissues a digestive fluid containing up to 200 mg. L^-1^ of natural proteins. Based on the properties of these natural bioreactors, we have evaluated the possibility to use carnivorous plants for the production of recombinant proteins. In this context, we have set up original protocols of stable and transient genetic transformation for both *Drosera* and *Nepenthes* sp. The two major drawbacks concerning the proteases naturally present in the secretions and a polysaccharidic network composing the *Drosera* glue were overcome by modulating the pH of the plant secretions. At alkaline pH, digestive enzymes are inactive and the interactions between the polysaccharidic network and proteins in the case of *Drosera* are subdued allowing the release of the recombinant proteins. For *D. capensis*, a concentration of 25 μg of GFP/ml of secretion (2% of the total soluble proteins from the glue) was obtained for stable transformants. For *N. alata*, a concentration of 0.5 ng of GFP/ml secretions (0.5% of total soluble proteins from secretions) was reached, corresponding to 12 ng in one pitcher after 14 days for transiently transformed plants. This plant-based expression system shows the potentiality of biomimetic approaches leading to an original production of recombinant proteins, although the yields obtained here were low and did not allow to qualify these plants for an industrial platform project.

## Introduction

Development of efficient and cost-competitive expression systems for the production of recombinant proteins constitutes a scientific but also an important economical challenge in the field of biotechnologies. Beside bacterial and mammal cells, plants are considered as interesting models for several reasons. As for mammals, plant cells can perform posttranslational modifications including glycosylation required for the optimal biological activity of many eukaryotic proteins. Plants are also particularly interesting because of the absence of potential pathogens such as mammalian viruses or oncogenic nucleic acids that could subsequently be transmitted to humans. Scientists and industry have taken advantage of these positive points and several plant recombinant protein based-products are currently undergoing clinical trials. For example, Elelyso produced in disposable bioreactors by an engineered carrot plant root cell line (ProCellEx^®^) and used for the treatment of Gaucher’s disease, was the first recombinant glycoprotein produced by a plant system and has been commercialized since 2012 (Mizukami et al., 2018). This plant cell system has also been used to produce Pegunigalsidase α (treatment of Fabry disease), Alidornase α (treatment of cystic fibrosis) and OPRX-106 (inflammatory bowel diseases) which have reached different clinical trials phases^1^. Another company, Greenovation GmbH, has developed a moss-based platform in which Moss-aGal (treatment of Fabry disease) is under clinical trial phase I investigation, whereas Moss-FH (human complement Factor H) and Moss-GAA (treatment of Pompe disease) are in preclinical development^2^.

The production and purification of pharmaceutical proteins goes through unavoidable steps such as tissue disruption, clarification of the crude extract, and purification of the product according to good manufacturing practices (GMP)(Fischer et al., 2012). These downstream processes represent up to 80% of the protein production costs and are, therefore, a key factor for commercial use of the production systems (Drossard, 2005). In comparison to microbial and mammalian cells, extraction steps from plants lead to the production of high amounts of cell scraps as well as many unwanted plant small contaminants, including secondary metabolites, chlorophyll and endogenous proteins. To bypass these time-consuming extraction and clarification steps, plant secretion-based systems have been considered. Several new generation platforms have been reported such as plant cell suspensions secreting recombinant proteins in the growth medium (*Nicotiana tabacum* BY-2 cells, ProCellEx^®^system, hairy roots,…) (Misaki et al., 2001; Häkkinen et al., 2014; Xu and Zhang, 2014; Tekoah et al., 2015; Cardon et al., 2018) or rhizosecretion of recombinant proteins from plants cultivated in hydroponic conditions (Madeira et al., 2016a,b).

Carnivorous plants are able to attract, trap, retain, kill, and digest preys (Juniper et al., 1989). They are found all around the world growing on nutrient-poor soils. They have established an original trait to circumvent the shortage of mineral nitrogen resources: their leaves have evolved to form traps for catching preys which turn to be an original source of nutrients. These preys are subsequently digested, allowing substantial recovery of nitrogen-rich molecules. *Drosera* and *Nepenthes* are two carnivorous plants genera able to produce and excrete out of their tissues a significant amount of digestive fluid. *Drosera* leaves are covered on their upper face by stalked glands secreting sticky and viscous digestive mucilage. *Nepenthes* leaves are differentiated in pitchers, the lower internal part being covered by glands secreting a digestive liquid (Juniper et al., 1989). Their digestive fluids contain proteins (especially digestive enzymes), a polysaccharide (responsible of the viscoelasticity proprieties), secondary metabolites (mainly antimicrobial compounds), and minerals salts (Juniper et al., 1989; Michalko et al., 2013; Kokubun, 2017; Krausko et al., 2017; reviewed by Miguel et al., 2018). Two major drawbacks have been identified in the use of these carnivorous plants as host of recombinant protein production: (1) the polysaccharidic network and (2) the proteases naturally present in the secretions.

The polysaccharide network is reported in *Drosera* and *Nepenthes* genus at different concentrations and is produced by stalked and sessile glands for *Drosera* and by pitted glands for *Nepenthes*. Several studies describe approximatively the chemical composition of this glue in *Drosera* genus. The mucilage contains mostly organic substances. Nearly 65% of them correspond to a polysaccharide (Kokubun, 2017) with a molecular weight between 2 × 10^6^ and 5 × 10^6^ Da (Rost and Schauer, 1977; Erni et al., 2008) and is composed by L-arabinose, D-xylose, D-galactose, D-mannose, and D-glucuronic acid in the molar ratio of 3.6:1.0:4.9:8.4:8.2 (Gowda et al., 1983). Another molecule described in the mucilage has been described as *myo*-inositol, a non-polysaccharide organic component. It has been highlighted between the polysaccharide strands and might acts as a cross linker *via* the formation of a hydrogen bond-network between the hydroxyl groups (Kokubun, 2017).

Concerning the native proteases present in secretion, several studies based on proteomic and genomic investigations determined the composition of enzymatic pool of *Nepenthes* digestive liquid. A complex mix of proteases was described in secretions such as aspartic proteases, cysteine proteases, serine carboxypeptidases and prolyl-endopeptidases (Athauda et al., 2004; Takahashi et al., 2005; Stephenson and Hogan, 2006; Hatano and Hamada, 2012; Kadek et al., 2014a; Lee et al., 2016; Rottloff et al., 2016). Few of them have been studied in detail and display an acid pH-dependent activity (Nepenthesin 1 and 2, Neprosins) (Athauda et al., 2004; Kadek et al., 2014b; Rey et al., 2016; Schräder et al., 2017).

To overcome the bottleneck linked to downstream process (DSP) costs, we have aimed to exploit this natural ability of carnivorous plants to secrete proteins and to assess the possibility to produce recombinant proteins from *Drosera* and *Nepenthes* plants. To achieve these goals, we have set up both a stable and a virus-based transient expression system for the production of recombinant proteins in the digestive fluid of these plants. We have also developed technical solutions to limit the impact of digestive proteases and polysaccharide matrix in the recovery of the recombinant proteins.

## Materials and Methods

### Plant Material and Virus

#### *Drosera* Plants *(D. capensis)*

*Drosera capensis* seeds were provided by Karnivore (Colmar, France) and conserved at 4°C. Seeds were sterilized by total immersion in a diluted commercial bleach solution containing 0.25% sodium hypochlorite for 5 min, and washed three times with sterile water. After a drying step on sterile paper, the seeds were sown on solid basal medium (capBM) composed by ½ Murashige and Skoog (MS) medium, 2-fold MS vitamin mixture, 2% (w/v) sucrose, 0.05% (w/v) casein hydrolysate, 50 mg/L citric acid, 100 mg/L ascorbic acid, 1 g/L polyvinylpyrrolidone and 0.7% (w/v) HP696 agar (Kalys, Bernin, France), and the pH was adjusted at 5.8. The seeds were incubated under a 16 h/8 h day/night photoperiod provided by natural white fluorescent lamps (160 μmol.m^-2^.s^-1^) at a temperature of 23°C. Seedlings were then transferred in the same fresh medium.

#### Nicotiana benthamiana

*Nicotiana benthamiana* seeds were sowed in compost and cultivated in controlled-environment room under 16 h/8 h day/night photoperiod with artificial light (70 μmol.m^-2^.s^-1^) at 26°C with 70% of relative air humidity. Three weeks-old plantlets were transplanted in individual pots and were used after 3–4 weeks for agroinfiltration experiments.

#### *Nepenthes* Plants *(N. alata)*

*Nepenthes alata* plant were purchased from Araflora^3^ and cultivated in heated greenhouses with natural light at 23°C with the relative air humidity of 75–85%.

#### Wild-Type TMV (wt-TMV)

Tobacco Mosaic Virus is multiplicated by rub inoculation of *N. benthamiana* leaves and tissues were collected 6 days post-inoculation. The TMV strain was provided by Prof Gilmer (IBMP Strasbourg – France).

### Genetic Constructions and *Agrobacterium* Preparation

To secrete GFP outside plant tissue, we used a GFP version with a signal peptide and without endoplasmic reticulum (ER) retention signal as in the case of native digestive enzymes. Consequently, *gfp* gene without ER retention signal was amplified from the binary vector pBin-m-gfp5-ER provided by Pr. Haselhoff (Division of Cell Biology, MRC Laboratory of Molecular Biology, Cambridge, CB2 2QH, United Kingdom). The primers were designed as following: gfp_Fw: 5′-GGATCCAAGGAGATATAACAATGAAGACTAATCTTTTTCTC-3′; gfp_Rev: 5′-TTATTTGTATAGTTCATCCATGCCATGTGTAATCCCAGC-3′). The amplification was done with Platinum Taq DNA Polymerase High Fidelity (Invitrogen^TM^, Thermo Fisher Scientific) and the PCR product was cloned into pCR8^®^/GW/TOPO^®^vector (Invitrogen^TM^, Thermo Fisher Scientific) as specified by the supplier. The coding sequence was subsequently transferred by recombination using LR Clonase II^TM^ (Invitrogen^TM^, Thermo Fisher Scientific) into binary vectors pGWB2-GW (AB289765.1) (Nakagawa et al., 2007) and pMW388-GW (JX971627.1) (Kagale et al., 2012) to obtain pGWB2-*gfp* for stable genetic transformation of *Drosera* and pMW388-*gfp* vectors (Supplementary Figure 1) for *Nepenthes* transient expression by recombinant virus inoculation. The recombinant plasmids were introduced into *Agrobacterium tumefaciens* C58C1Rif^R^ using the freeze-thaw method (Chen et al., 1994) and selected in solid YEB (10 g/L Beef Extract, 5 g/L Yeast Extract, 10 g/L peptone, 15 g/L sucrose, 0.5 g/L MgSO_4,_ 20 g/L at pH 7.2) supplemented with 100 mg/l rifampicin, 100 mg/L carbenicilin and 30 mg/l kanamycin at 28°C during 2–3 days.

### Stable Genetic Transformation of *D. capensis*

#### *Agrobacterium* Suspension Preparation

The *A. tumefaciens* strain C58C1Rif^R^ transformed with binary vector pGWB2-*gfp* and pMW388-*gfp* were cultured in liquid YEB medium containing 100 mg/l rifampicin, 100 mg/L carbenicilin and 30 mg/l kanamycin at 28°C during 2 days at 180 rpm. Four hours before plant transformation, 100 μM of acetosyringone was added to bacteria cultures to activate the *vir* genes. The bacteria were pelleted by centrifugation for 15 min at 5000 × *g*, washed two times with fresh YEB medium to remove antibiotics and re-suspended at a cell density of OD_600_ 0.8 ± 0.1 in liquid capBM medium.

#### *Agrobacterium* Mediated-Transformation of *Drosera*

The protocol of genetic transformation *D. capensis* developed for this work is based on the method developed by Hirsikorpi et al. (2002). Leaf explants of 6 months old *in vitro* plants were wounded with a sterile needle and completely immerged in *Agrobacterium* suspension for 10 min. Explants were transferred on solid capBM medium and cultivated at 23°C under a 16 h/8 h day/night photoperiod for co-culture step. After 3 days, explants were washed with liquid capBM medium supplemented by 200 mg/L cefotaxime for 10 min. They were then transferred on solid capBM medium with 60 mg/L kanamycin and 200 mg/L cefotaxime supplemented by 5 μg/L 6-benzylaminopurine.

#### Generation of Transgenic Plants

After 2 months, regenerated plantlets were separated from initial explants and transplanted on solid BM medium supplemented by 5 μg/L 6-benzylaminopurine, 200 mg/L cefotaxime and 300 mg/L kanamycin for 4 months. When plants were 4–5 cm high, they were rooted in solid BM medium supplemented by 200 mg/L cefotaxime, 300 mg/L kanamycin and 250 μg/L indole-3-butyric acid for 2 months.

#### *Ex vitro* Acclimation

Rooted plants were acclimated in greenhouse under natural light at a temperature of 23°C and a relative air humidity of 75–85%. The substrate was composed by 1/3 of sphagnum and 2/3 of blond peat.

#### Obtention of T1 Progeny

Because of the risk to obtain chimeric transgenic plants due to their multicellular origin, the production of recombinant proteins was based on T1 generation obtained 4 months after acclimation. Seeds of potentially chimeric transgenic plants were collected, sterilized and cultivated on solid BM medium supplemented with 100 mg/L kanamycin for 4 months. Then, the selected plants were rooted and acclimated as described above.

### *Nepenthes* Transient Expression by Recombinant Virus Inoculation

#### Multiplication of Recombinant TMV in *N. benthamiana* by Agroinfiltration

*Agrobacterium* suspension was prepared as described above. The bacteria pellet was resuspended in infiltration buffer (10 mM MES, pH 5.6). The OD_600__nm_ was adjusted at 0.5. The *Agrobacterium* suspension was injected using a syringe into the abaxial side of *N. benthamiana* fully expanded leaves. Infiltrated plants were cultivated under 16 h/8 h day/night photoperiod at 26°C with 70% of relative air humidity for 14 days. Leaves were then harvested at 5–7 days post-infiltration and stored at -80°C.

#### Wild-Type and Recombinant Virus Inoculation in *Nepenthes* Leaves

Infected *N. benthamiana* leaves by wt-TMV and agroinfiltrated *N. benthamiana* leaves for recombinant virus multiplication were ground in NaPi buffer (0.5 M Na_2_HPO_4_) at pH 8 in 1:4 ratio using a mortar and pestle. The crushed tissues were supplemented with 1% (w/v) Celite 545 AW (Sigma-Aldrich) and used for rub inoculation of adaxial face of *N. alata* mature leaves attached to a just opened pitcher. Before inoculation, native secretions of each pitcher were removed, cleaned with water and replaced by 30-40 ml phosphate buffer (137 mM NaCl, 2.7 mM KCl, 8.1 mM Na_2_HPO_4_, 1.5 mM KH_2_PO_4_ at pH 7.4). Pitcher were bagged to keep high humidity and to limit contaminations. Fifteen minutes after inoculation, the leaves were rinsed with tap water to remove the residual sap and celite. Leaves were wrapped, and plants were returned to the standard growth conditions.

### Molecular Analysis

#### PCR Based Molecular Characterization of the *Drosera* Transgenic Plants

Genomic DNA of *Drosera* was extracted from leaves according to Biteau et al. (2012). Genes of interest integrated on genomic DNA was detected thanks to Master Mix from Thermo Fisher Scientific. The primers designed to amplify housekeeping gene 18S (AY096118.1) were Dcap18S1_Fw: 5′-CGTGCAACAAACCCCGAC-3′ and Dcap18S1_Rev: 5′-TGCGCGCCTGCTGCCTT-3′. The primers designed to amplify *gfp* gene were gfp1_Fw: 5′-ATCCTCGGCCGAATTCAGTAAAGG-3′ and gfp1_Rev: 5′-AGTTCATCCATGCCATGTGTAATCCC-3′.

#### RNA Extraction and RT-PCR From *Drosera* and *Nepenthes* Tissues

Total RNA was extracted from *Drosera* and *Nepenthes* tissues using the Spectrum^TM^ Plant Total RNA kit (Sigma-Aldrich) according to the manufacturer’s instructions. To efficiently remove genomic DNA, RNA was treated with the Turbo^TM^ DNA Free Kit (Ambion, Thermo Fisher Scientific). SuperScriptIII One-step RT-PCR Kit (Invitrogen^TM^, Thermo Fisher Scientific) was used to detect transcripts of interest from 50 ng of total RNA. For *Drosera*, the primers designed to amplify 18S transcripts were Dcap18S2_Fw: 5′-GGGTTCGCCCCGGTTGCTCTGATGATT-3′ and Dcap18S2_Rev: 5′-GGGCCGAGACGATAGGTGCACAC-3′, the primers designed to amplify nptII transcripts were nptII_Fw: 5′-GGATTGCACGCAGGTTCTCCGGCCG-3′ and nptII_Rev: 5′-TGGCCAGCCACGATAGCCGCGCTG-3′ and the primers designed to amplify *gfp* transcripts were gfp2_Fw: 5′-GGATCCAAGGAGATATAACAATGAAGACTAATCTTTTTCTC-3′ and gfp2_Rev: 5′-GTCGTGCCGCTTCATATGATCTGGGTATC-3′. For *Nepenthes*, the primers designed to amplify movement protein transcripts of TMV were TMV-MP_Fw: 5′-ATGGCTCTAGTTGTTAAAGGAAAAGTGAATATC-3′ and TMV-MP_Rev: 5′-CACATTTCTAATATTAACTAAAACTTGCCAG-3′, the primers designed to amplify capsid protein transcript of TMV were TMV-CP_Fw: 5′-ATGTCTTACAGTATCACTACTCCATCTCAG-3′ and TMV-CP_Rev: 5′-TCAAGTTGCAGGACCAGAGGTCC-3′ and the primers designed to amplify *gfp* transcript were gfp3_Fw: 5′-ATGAGTAAAGGAGAAGAACTTTTC-3′ and gfp3_Rev: 5′-GTCGTGCCGCTTCATATGATCTGGGTATC-3′.

### Analysis of Protease Activity and Recombinant GFP in the Secreted Liquid

#### Treatment of Secretions

##### Collection of secretions and recovery of recombinant proteins from *Drosera* glue

Because *Drosera* plants produce a limited amount of viscous liquid, we decided to collect two series of secretions from five non-chimeric plants. These 8 months-old plants presented the same development stage and were previously obtained from independent transformation events. The harvest was realized by immerging *D. capensis* leaves in a buffer (Tris HCl 50 mM pH 7.5) during 5 min under gentle agitation and the leaves were rinsed with distilled water after the first harvest. In total, a volume of 200 ml of buffer was recovered and subsequently filtrated though a Whatman^®^filter with 2.5 μm of porosity. To capture recombinant proteins into *Drosera* mucilage, 1 ml of Anion Exchange chromatography resin (HyperCel STAR AX, 20197-026, PALL) was added for 200 ml of sample. The mix was agitated at 4°C for an overnight and the resin was recovered and washed with 5 ml of washing buffer (Tris HCl 50 mM pH 7.5, NaCl 5 mM). Recombinant proteins were eluted three times with Tris HCl 50 mM pH 7.5 with increasing concentrations of NaCl from 0.2 M, 0.5 M to 1 M.

##### Concentration of *Nepenthes* secretions by tangential flow filtration and sample preparation

Two batches of *Nepenthes* secretions were prepared from four pitchers attached to four leaves inoculated independently by recombinant TMV. The two batches were pooled, filter sterilized (0.2 μm) and concentrated about 20 times by tangential flow filtration (Cogent Microscale, Millipore) thanks to 5K Minimate capsule with Omega membrane (PALL). Recombinant proteins were precipitated overnight at -20°C with 4 volumes of acetone. The samples were then centrifuged at 12,000 × *g*, for 30 min at 4°C. The pellets were dried 2 h at 37°C.

#### Analyses of Carnivorous Plant Secretions

##### Zymograms with *Nepenthes* secretion

Pellets were solubilized in 30 μL of Tris/Glycine buffer x1 (TrisHCl 25 mM, glycine 192 mM, pH 8.3) and 10 μl of native buffer (62.5 mM TrisHCl pH 6.8, SDS 2% w/v, 0.001 % bromophenol blue). The samples were separated by SDS-PAGE using a 10% polyacrylamide gel containing 0.1% casein. The gel was washed 2 times for 15 min with renaturation buffer (Triton X-100 2.5% at pH 3 or 8) to remove SDS and was incubated overnight at 37°C in an activation buffer. To test the impact of pH conditions in *Nepenthes* protease activity, a set of activation buffers was constituted: for pH 2, 9.8 mM citric acid and 0.24 mM disodic phosphate; for pH 3, 8 mM citric acid and 4 mM disodic phosphate; for pH 4, 1.6 mM sodic acetate and 8.3 mM acetic acid; for pH 5, 6.8 mM sodic acetate and 3.2 mM acetic acid; for pH 6, 3.7 mM citric acid and 12.5 mM disodic phosphate; for pH 7, 1.9 mM citric acid and 16.2 mM disodic phosphate; for pH 8, 12 mM boric acid and 4.5 mM HCl; for pH 9,16.7 mM boric acid and 1.6 mM HCl.

##### SDS-PAGE and western-blot analyses of *Nepenthes* secretions

Pellets were solubilized in 30 μL of Tris/Glycine buffer x1 (TrisHCl 25 mM, glycine 192 mM, pH 8.3) and 15 μL of denaturating buffer (0.313 M Tris-HCl pH 6.8 at 25°C, 10% SDS, 0.5% bromophenol blue, 50% glycerol, 2 M dithiothreitol). Proteins were heated at 95°C for 10 min. The samples were loaded on MiniPROTEAN^®^TGX^TM^ precast gels, 10% polyacrylamide (50 μL, 10-well; Bio-Rad). The separated proteins were blotted on a polyvinylidene fluoride membrane (Membrane PVDF 0.45 μm Amersham^TM^ Hybond^TM^ P, GE Healthcare) and GFP was detected thanks to a rabbit primary polyclonal antibody anti-GFP (NB600-310, Novus Biological) at 1:5000 dilution in PBS (137 mM NaCl, 2.7 mM KCl, 8.1 mM Na_2_HPO_4_, 1.5 mM KH_2_PO_4_, pH 7,4) and to a secondary anti-rabbit antibody conjugated to alkaline phosphatase activity (Cat#A0418-1ML, Anti-Rabbit IgG (whole molecule)–Alkaline Phosphatase antibody produced in goat, Sigma-Aldrich) diluted to 1:6000 in PBS. Revelation was performed with NBT/BCIP (Promega, United States) as substrate.

##### GFP quantification on *Nepenthes* secretions

Enzyme-linked immunosorbent assay (ELISA) was used to quantify the amount of GFP present in the secretions. Pellets were solubilized in 100 μL of PBS and coated overnight on plates (microplates Greiner BioOne Cat #655051) at 4°C. GFP standard (Recombinant *Aequorea victoria* GFP, AR09180PU-N, Acris) was used to generate a standard curve over a range between 0.16 and 10 μg/ml. A rabbit primary polyclonal antibody anti-GFP (Cat# NB600-310, Novus Biological) was used at 1:2000 dilution. A secondary anti-rabbit antibody conjugated to HRP [Peroxidase AffiniPure Goat Anti-Rabbit IgG(H+L) 111-035-003, Jackson Immuno Research] was used at 1:6000 dilution for detection, using TMB-ELISA (Thermo Fisher Scientific) as substrate. The plate was developed for 15 min and the reaction was stopped with 2M H_2_SO_4_.

##### GFP quantification on *Drosera* glue

GFP quantification was performed by fluorometry and the concentration was determined by comparing values of the protein extracts to a standard curve made with a standard of recombinant GFP (Recombinant *Aequorea victoria* GFP, AR09180PU-N, Acris).

##### Total soluble proteins (TSP) quantification in secretions

To quantify TSP in secretions, we used Qubit^TM^ Fluorometer (Invitrogen^TM^, Thermo Fisher Scientific) with Qubit^TM^ protein assays kit (Invitrogen^TM^, Thermo Fisher Scientific) according to supplier instructions.

## Results

### Production of Recombinant Proteins From *Drosera* Glue

#### Molecular Characterization of Transgenic *D. capensis*

Establishment of an *Agrobacterium*-mediated transformation protocol and subsequent regeneration of transgenic plants require setting up several parameters such as an optimal hormonal balance adapted to plant regeneration, the selection of transformed cells and parameters linked to transfection of T-DNA from agrobacteria to plant cells. Such experimental conditions were described by Hirsikorpi et al. (2002) for *D. rotundifolia* but needed to be revisited for *D. capensis*. Thus, several parameters are completely different from those of *D. rotundifolia*. To regenerate *D. capensis* plantlets from leaf explants we only used low quantity of BAP (0.005 mg/L) in contrary to *D. rotundifolia* that needed a combination of auxin and cytokinin (0.45 mg/L BAP and 0.372 mg/L 1-Naphthaleneacetic acid). The selection of transformed plant was performed in presence of 60 mg/L of kanamycin at the explant level and 300 mg/L after bud apparition. These different steps were conducted at a continuous kanamycin concentration for *D. rotundifolia* (400 mg/L). Finally, plantlets were rooted in 0.27 mg/L IBA while no hormone was added for *D. rotundifolia*.

**Figure 1:**
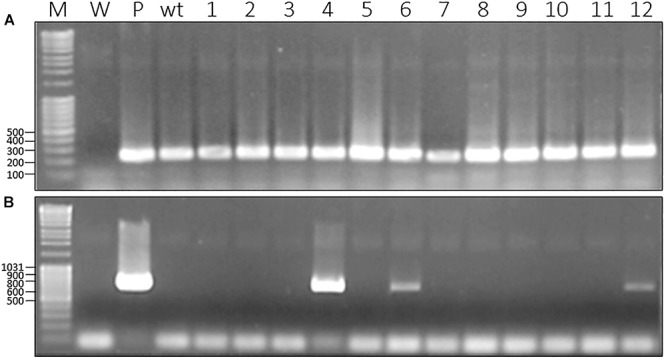
Molecular characterization of *gfp*-transformed *D. capensis* from T1 generation produced by chimeric transgenic plants. **(A)** PCR with primers specific to 18S gene as housekeeping gene (270 bp); **(B)** PCR with primers specific to *gfp* gene (720 bp); M, Marker; W, water as negative control; wt, wild-type; P, plasmid harboring the gene as a positive control; 1–12, non-chimeric plants issues from T1 generation.

To avoid working on plants potentially chimeric, we produced seeds from self-pollinated transgenic plants. However, we didn’t determine whether the transgenic lines were heterozygous or homozygous, a characteristic which might affect the amount of recombinant protein produced. The resulting plants were screened for the presence of the *gfp* coding sequence using a PCR approach with specific primers. An expected 720 bp amplicon could be highlighted in some plants (Figure 1). A series of 23 plants was selected, based on the occurrence of the expected PCR product, from a group of 50 plants previously obtained by direct regeneration of transformed calluses. Each of these 50 plants corresponded to independent transformation events.

**Figure 2:**
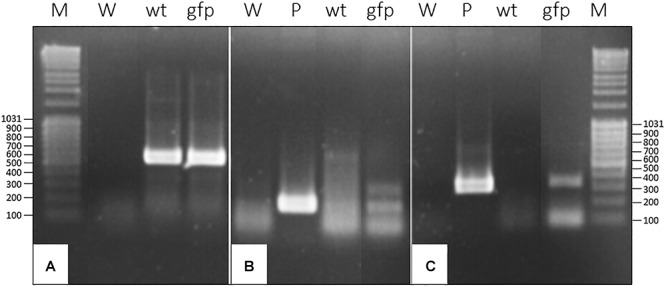
RT-PCR on RNA extracted of *gfp*-transformed *D. capensis* plant coming from T1 generation produced by chimeric transgenic plant. **(A)** RT-PCR with primers specific to 18S ribosomal RNA gene as housekeeping gene (467 bp); **(B)** RT-PCR with primers specific to *nptII* (199 bp); **(C)** RT-PCR with primers specific to *gfp* (329 bp); M, Marker; W, water; wt, RNA from wild-type plant; P, plasmid; gfp, RNA from non-chimeric *gfp* transformed-plant.

Beside the insertion of the T-DNA in the genome, we also assessed the expression of *gfp* and *nptII* using a RT PCR approach on RNA extracted from different recombinant plants. In addition to the 329 bp amplicon corresponding to the *gfp*, we could also amplify a 199 bp amplicon corresponding to the *nptII* selection marker (Figure 2).

#### Highlighting the Production of Recombinant Proteins Outside Plant Tissues in *Drosera* Secretions

**Figure 3:**
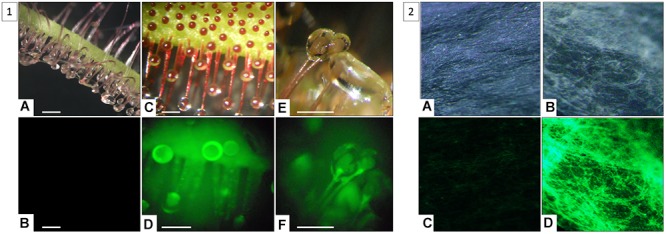
Highlighting of GFP secreted by tentacles **(1)** outside plant tissues into secretions of *gfp*-transformed *D. capensis* **(2). (1)** Observation of *D. capensis* wild-type tentacles **(A,B)** (scale = 1 mm) and *gfp*-transformant tentacles **(C–F)** (scale = 0.5 mm) under natural light **(A,C,E)** and under UV-light **(B,D,F)**; **(2)** Observation of glue absorbed directly on Whatman paper from wild-type plant **(A,B)** and *gfp*-transformant **(C,D)** and under exposition to natural light **(A,C)** and UV-light **(B,D).**

Glue from wild-type *D. capensis plant* and *gfp*-transformant were collected on filter paper and visualized under exposition to UV-light (395 nm). In contrary to wild-type (Figure 3-1A,B,3-2A,B), transformed plants displayed fluorescence at the tips of tentacles (Figure 3-1C–F) and into secretions (Figure 3-2C,D). These observations made evidence that GFP was produced into the leaf tissues but also secreted into the mucilage thanks to glandular hairs.

The digestive fluids of *Drosera* plants is composed of a highly viscous and elastic mucilage making the collection of recombinant proteins not trivial. To overcome this problem, we developed a two-step strategy. First, we showed that the binding of the polysaccharidic network can be relaxed by dilution and alkalinization with a pH 7.5-adjusted buffer. Then, to catch and concentrate the released proteins, we used an anion-exchange chromatography resin which binds to negatively charged molecules such as GFP displaying an isoelectric point at 5.8. This strategy led us to recover functional and concentrated GFP.

#### Quantification of Recombinant Protein Production by *Drosera* Genus

The quantification of GFP production by *Drosera* was performed by fluorimetry measurements using commercial GFP to establish a standard curve. Given that glue of *Drosera* presents an auto-fluorescence due to some compounds secreted in this matrix especially secondary metabolites, this native fluorescence was subtracted of signal obtained from glue issue from transgenic plants. Thus, we have estimated the production of recombinant protein in *Drosera* glue at 26.07 ± 2.73 μg of GFP/ml of secretions. This represents at 26.07 ± 2.73 μg of GFP/ml of secretions for five plants. So, one plant can produce 0.2 ± 0.02 μg of GFP with the relative yield of about 2% of TSP from the glue.

### Production of Recombinant Proteins Into *Nepenthes* Digestive Liquid

#### Molecular Characterization of Inoculated *Nepenthes* by Recombinant TMV

##### Capacity of TMV to infect *Nepenthes*

**Figure 4:**
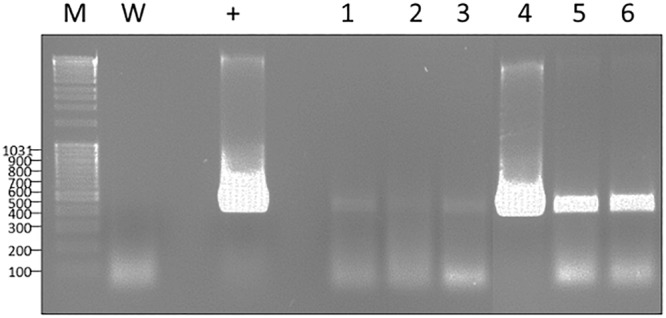
RT-PCR exhibiting the capacity of TMV to infect and to move systemically into *N. alata* plant in 14 days. Use of specific primers to movement protein gene (502 bp); M, Marker; W, water; +, RNA from *N. benthamiana* infected by TMV; 1, 2, 3, RNA of different parts of *N. alata* plant inoculated with safety *N. benthamiana* extract; 4–6, RNA of different parts of *N. alata* plant inoculated with *N. benthamiana* extract infected by TMV; 1 and 4, RNA from inoculated leaf; 2 and 5, RNA from pitcher attached to inoculated leaf; 3 and 6, RNA from neighboring leaf. Parts of gels separated by a space were grouped together and lined up to facilitate the visualization of results. Any changes touching the quality of picture were applied to every pixel of each part.

Tobacco mosaic virus is one of the most extensively studied plant viruses and has consequently become a natural choice for vector development. As a preliminary experiment, we investigated the capacity of wt-TMV to infect *Nepenthes* tissues. Four mature leaves attached to just opened pitcher were inoculated per plant, with a *N. benthamiana* crude extract infected wild-type virus. 14 days post-inoculation (dpi), total RNA from different parts of plants were extracted and the presence of virus was assessed by RT-PCR. Despite the absence of any symptom, we could successfully amplify a specific 502 bp amplicon, corresponding to the gene encoding the movement protein transcripts, in the inoculated leaves but also in the pitcher tissues attached to the inoculated leaf, and in a neighboring leaf (Figure 4). This result clearly makes evidence that *N. alata* is a host plant of TMV which might therefore be used as a tool for realizing transient expression.

##### Systemic expression of transiently expressed genes in *Nepenthes* tissues

**Figure 5:**
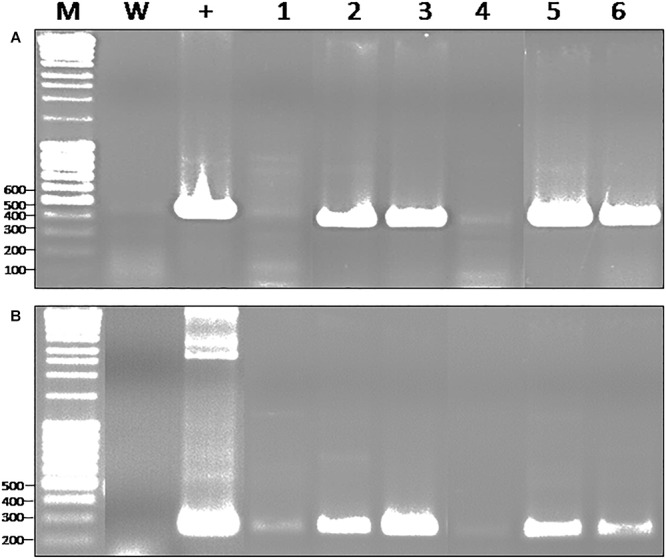
RT-PCR exhibiting the capacity of recombinant TMV to infect and to move systemically into *N. alata* plant in 14 days. **(A)** Use of specific primers to TMV capsid protein gene (480 bp); **(B)** Use of specific primers to *gfp* (246 bp); M, Marker; W, water; +, RNA from *N. benthamiana* infected by TMV-*wt* **(A)** and agroinfiltrated by pMW388-*gfp* plasmid **(B)**; 1 and 4, RNA from inoculated leaf with safety *N. benthamiana* extract at 5 days (1) and 14 days (4); 2 and 5, RNA from inoculated leaf with *N. benthamiana* extract infected by TMV-*wt* and agroinfiltrated by pMW388-*gfp* at 5 days (2) and 14 days (5); 3 and 6, RNA from pitcher attached to inoculated leaf with *N. benthamiana* extract infected by TMV-*wt* and agroinfiltrated by pMW388-*gfp* at 5 days (3) and 14 days (6) post inoculation. Parts of gels separated by a space were grouped together and lined up to facilitate the visualization of results. Any changes touching the quality of picture were applied to every pixel of each part.

For producing GFP in *Nepenthes* tissues, we introduced the corresponding gene in the pMW388 vector leading to a recombinant TMV lacking the gene encoding the capsid protein (CP) but including the *gfp* coding sequence. This recombinant virus was multiplied in *N. benthamiana* leaves by using an agroinfiltration approach. A crude extract was produced from the infected *N. benthamiana* tissues and used to inoculate *Nepenthes* leaves. Since the CP is necessary for a systemic infection, we realized a co-inoculation of the engineered virus with crude extracts from *N. benthamiana* leaves infected with wild-type TMV providing the necessary CP. Four mature leaves per plant, attached to just opened pitcher, were inoculated, and the native secretions of each pitcher were replaced by phosphate buffer at pH 7.4. Five and 14 dpi, tissues of inoculated leaves and the bottom part of the correspondingly attached pitcher containing secretions were collected. A RT-PCR performed on RNA extracted from these tissues showed the expression of *cp* gene (coming from the wild-type TMV), and *gfp* gene (coming from the recombinant virus), whereas no transcripts could be highlighted in non-inoculated plants (Figure 5). This result makes evidence that the engineered TMV can infect *Nepenthes* tissues and that the CP produced by the wt-TMV can help *in trans* for its movement in the whole inoculated plant.

#### Recombinant Protein Production in *Nepenthes* Secretions

##### Development of a strategy to protect recombinant proteins from secreted digestive enzymes

**Figure 6:**
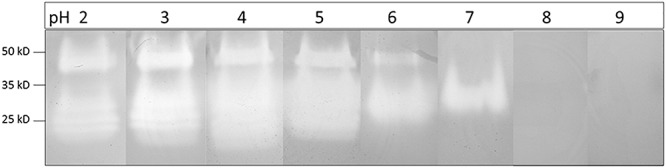
Zymograms under *N. alata* secretions using polyacrylamide gel containing casein incubated between pH 2 and pH 9. Each pH modality was established in separated SDS-PAGE. Zymogram profiles at each pH value separated by a space were grouped together.

Given the high proteolytic activity of *Nepenthes* digestive fluid, it was necessary to set up a strategy to protect recombinant proteins secreted in this unfavorable environment. Several studies described that some of the proteases present in the digestive fluids were acid pH-dependent (Athauda et al., 2004; Rey et al., 2016). We confirmed these data with casein containing zymograms realized on *N. alata* secretions. The gels were incubated in buffer ranging from pH 2 up to pH 9 (Figure 6). Our results showed that *Nepenthes* secretion exhibited a maximum proteolytic activity at pH 3-4 and was inhibited after pH 7. Several lysis spots could be observed highlighting a degradation of casein, probably by several proteases with different sizes.

**Figure 7:**
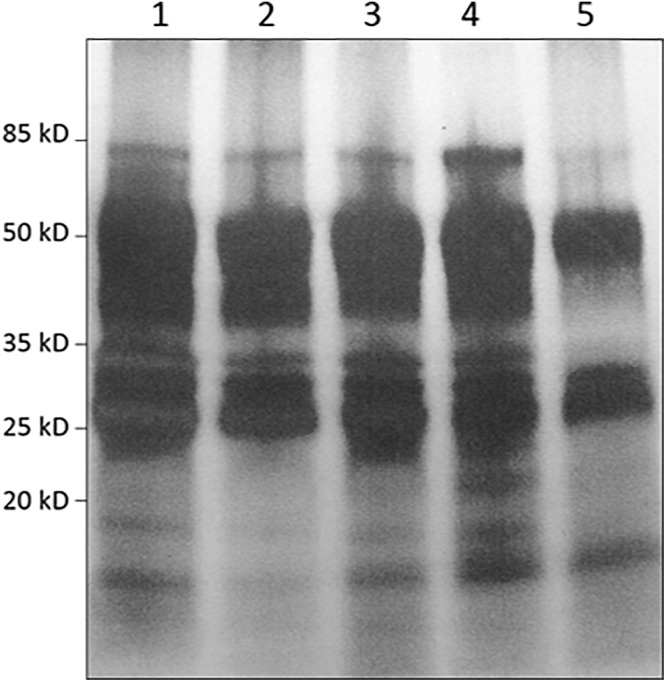
Highlighting of the capacity of *Nepenthes* pitcher to renew its digestive pools after several collects and replacements of liquid by an alkaline buffer *via* SDS-PAGE analysis. (1) Secretion pool after opening pitcher and before the first replacement; (2) Secretion pool 3 days after the first replacement of digestive liquid by a buffer; (3) Secretion pool 3 days after the second replacement; (4) Secretion pool 3 days after the third replacement; (5) Secretion pool 3 days after the fourth replacement.

We assumed that a way for preserving the recombinant protein might be to increase the pH in the pitcher. We realized a time-course experiment for the production of these proteases. We replaced the native secretion fluid by an alkaline buffer, and we collected and analyzed it 3 days after. We made four replacements of liquid for the same set of pitchers every 3 days. This experiment showed that the digestive protein pool is reconstituted 3 days after removing the liquid pitcher because we obtained similar protein profiles (Figure 7) and similar concentrations of TSP, respectively 45.2 ± 9.7, 33.75 ± 9.2, 44.0 ± 20, 51.75 ± 4.3, and 45.2 ± 15.7 μg of TSP/ml of secretion at each harvest (experiments performed with 4 pitchers). It also demonstrates that the replacement of the native fluid doesn’t interfere with the production of proteins.

##### GFP production by *Nepenthes* secretions

**Figure 8:**
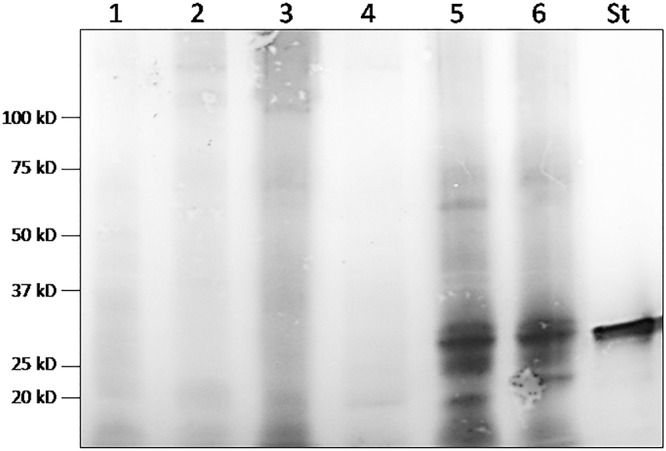
Western-blot analyses of secretions from one *N. alata plant* inoculated with agroinfiltrated *N. benthamiana* with empty vector (1 and 4) and from two *N. alata* plants inoculated with *N. benthamiana* extract infected by TMV and agroinfiltrated by TMV-ΔCP-*gfp* (2, 3, 5, and 6) at 5 days (2 and 3) and 14 days (5 and 6) post inoculation (St, GFP standard).

For assessing the production of GFP in pitchers, engineered and wild-type TMV were co-inoculated to leaves connected to a mature pitcher for which the digestive fluid was replaced by a phosphate buffer at pH 7.4 prior to the inoculation. We collected the secretion 5 and 14 dpi and measured the pH, confirming this way its stability. The secretions were pooled, concentrated by tangential filtration and precipitated with acetone. The protein pellets were analyzed by western-blot using an anti-GFP serum (Figure 8). Whereas no GFP could be detected 5 dpi, a 27 kDa protein was produced 14 dpi. This analysis confirmed that GFP can be efficiently produced at the same time than the digestive protein pool. It also showed that the alkalization of secretions can, at least, limit or slow down degradation of the recombinant protein. Indeed, several low size bands can be observed, constituting potentially an evidence of partial degradation by digestive enzymes.

##### Quantification of recombinant GFP protein production by *Nepenthes* model

An ELISA quantification test was established by using commercial GFP as a standard. This quantitative assay showed that four pitchers attached to four leaves inoculated independently by recombinant TMV can produce 48.2 ± 1.4 ng of GFP in 14 days, namely 11.95 ± 0.45 ng of GFP/pitcher. The GFP was measured at an initial concentration of 0.595 ± 0.025 ng of GFP/ml secretions which represents on average 0.5% of TSP from the secretions.

## Discussion

Producing recombinant proteins for pharmaceutic purposes by plant platforms is an attractive prospect for many well-documented reasons. Plants can be easily cultivated at affordable cost at a large scale. Plants are also interesting because of their ability to correctly fold, assemble and process complex proteins. So far, no contamination by mammalian viruses or pathogens has been reported and using plants also avoid any ethical issues in comparison to transgenic animals.

Since the first reports dedicated to the construction of transgenic tobacco in the 1980s, plants have been largely used for the production of proteins for research purposes (Vyacheslavova et al., 2012) and were considered for the production of pharmaceuticals at the industry level. During these few decades, scientists demonstrated that plants could produce adequate quantities of functional human proteins. The comprehension of the gene silencing processes and the use of plant virus-based vectors opened some new tracks in the years 2000 (Pumplin and Voinnet, 2013). With these methods mostly based on agroinfiltration, large amounts of recombinant of proteins can be realized within a few days increasing the interest of plant systems in this field. Nowadays, both stable and transient systems are used for the production of such proteins (Paul and Ma, 2011; Sheshukova et al., 2016).

To access to any pharmaceutical market, proteins need to be purified with contaminants removed to acceptable levels. The downstream processing starts with the harvesting of plant biomass followed by a tissue disruption (Menkhaus et al., 2004; Wilken and Nikolov, 2012; Buyel and Fischer, 2014). The subsequent extraction step aims to release the recombinant protein from the plant material into an aqueous buffer. The main problem for plant material is the presence of coproducts such as endogenous cell proteins, intrinsic plant fibers, oils, polyphenols, chlorophylls or organic acids. Furthermore, plant solids are generally in higher concentration, wider in size range, and denser than conventional cell culture debris. Some of these contaminants can be removed through clarification using centrifugation and/or filtration (Buyel et al., 2015). These preliminary extraction and clarification steps are quite costly in comparison to the direct purification used for microbial and mammalian cell systems (Buyel et al., 2015) and represent one of the most important drawbacks of the plant expression system.

To overcome this problem, scientist have focused on plant secreted based-systems that have been described in literature. In the case of rhizosecretion or cell cultures, the proteins can be released in the culture medium. Some of these systems have been described for several proteins and were used at the industrial scale (Tekoah et al., 2015). The carnivorous plant-based system described in this report can be considered as a secretion based-system. *Drosera* have large leaves recovered by glandular hairs and the bottom of the *Nepenthes* pitchers is recovered by a network of multicellular glands [between 3.72 glands/mm^2^ and 8.87 glands/mm^2^ in *N. alata* (Wang et al., 2009)]. In this work, we demonstrated that, thanks of these specialized organs, recombinant protein follows the same way as native proteins, and are excreted outside plant tissues. Recombinant proteins can be secreted and easily recovered by washes of *Drosera* tentacles or by emptying *Nepenthes* pitchers. The heterologous proteins are however mixed with 20–30 endogenous proteins, antimicrobial compounds, mineral nutrients and a polysaccharidic macromolecule (reviewed by Miguel et al., 2018).

Removing the polysaccharide represents the main challenge for purifying the secreted recombinant protein. This macromolecular network is responsible of the viscoelasticity proprieties of secretions and is a key element for the capture of prey used by carnivorous plants (Adlassnig et al., 2010; Król et al., 2012). The shear viscosity of the mucilage is about 10^2^Pa.s (Erni et al., 2008), the maximum viscosity is reported at pH 5 and can decrease when pH is raised or lowered (Rost and Schauer, 1977; Kokubun, 2017). These properties are helpful for purifying the recombinant proteins stacked in the glue. The alkalization realized by washes of the leaves with a buffer, significantly decreased the viscosity of the glue by disrupting the polysaccharidic network without degradation of the target protein. The alkaline pH also changed the electric charge of the protein that became negative and could therefore be purified with an anionic resin.

For *Nepenthes*, the exact composition of the biopolymer remains to be elucidated although the *Nepenthes* fluid could exhibit a similar acidic polysaccharide mucilage than *Drosera* (Gaume and Forterre, 2007). Its viscoelasticity strength of 1.4.10^-2^ Pa.s is less important than *Drosera* and could be linked to a lower quantity of secreted polysaccharide. *N. alata* exhibits a non-viscoelastic apparently water-like fluid in contrary to some *Nepenthes* species producing viscous secretions as *N. rafflesiana* or *N. aristolochioides*, (Gaume and Forterre, 2007). The purification of the proteins might therefore be easier at large scale in this used in our study.

Unwanted degradation of recombinant proteins by endogenous proteases is another drawback of plant-based heterologous production systems (Pillay et al., 2014). These enzymes are involved in a multitude of processes from cellular to whole organism level, but the exact functions and targets of most of them are still unknown (van der Hoorn, 2008). In the case of a production inside plant tissues, proteolysis events might occur into different cellular compartments and during the extraction step (Sharp and Doran, 2001; Drake et al., 2003; Niemer et al., 2014; Pillay et al., 2014). These proteases represent more than 10% of the extracellular proteins (Albenne et al., 2013). Several strategies have been described in literature to counteract these enzymes such as (1) using protein stabilization agents as gelatin, BSA (Bovine Serum Albumin) or other low value proteins acting as competitive substrates for proteases (Drake et al., 2003; Huang and McDonald, 2012), (2) using inhibitor cocktails, (3) coexpressing protease inhibitors or (4) inhibiting the expression through a gene silencing approach (Komarnytsky et al., 2006; Kim et al., 2008; Goulet et al., 2010; Robert et al., 2013; Jutras et al., 2016). According to Lallemand and collaborators, the nature of the proteases depends on the production systems but also the developmental stages, the culture medium or the plant species (Lallemand et al., 2015). Thus, the obtention of ‘omics’ data in a given production system is an interesting tool to perform the identification of proteases potentially dangerous for the target protein (Rottloff et al., 2016).

In carnivorous plant-based system, *Nepenthes* secretion exhibited maximum proteolytic activity at pH 3–4 but was inhibited beyond pH 7 as described by Rey et al. (2016) and confirmed by our experiments. The native pitcher fluid has a pH of 2.8 after opening in presence of prey which is favorable for activating a proteolytic activity and to digest insects. Changing the pH to 8 completely inhibited the proteolysis events in the digestive liquid without disturbing the secretion process of the digestive glands. The access to the *Nepenthes* pitcher is therefore quite comfortable for controlling the degradation of the recombinant protein.

This development of carnivorous plant-based expression systems requested to develop original protocols to successfully express gene into plant tissues. A first approach consists of generating stable transgenic plants. The first plant selected for this study was *D. capensis*, the Cape sundew which has longer leaves than the common sundew for the production of large amounts of glue (Hirsikorpi et al., 2002). For *D. capensis*, an *Agrobacterium*-mediated transformation protocol was developed, based on Hirsikorpi et al. (2002) work done on *D. rotundifolia* with readjustment of several parameters. This method necessitates 18 months to regenerate transformed plants dedicated to production of recombinant proteins, which is a long time scale in a research program. To reduce the production time, an alternative technique of genetic transformation was successfully developed, based on a floral dip protocol. This type of *in planta* transformation, consist in agro-infecting male or female gametophytes present in flowers and is used with several species like *Arabidopsis thaliana* (Clough and Bent, 1998), *Raphanus sativus* (Curtis and Nam, 2001), or wheat (Agarwal et al., 2009). It allows the obtention of an important amount of transformed seeds and avoid the possibility of chimeric plants since each plant is theoretically derived from an embryo of single cell origin. A critical parameter for success is the developmental stage of the flower at the time of inoculation with *Agrobacterium* (Clough and Bent, 1998). Given that floral stalks of *Drosera* exhibit flowers at different development stages, some of them are likely to be transformed by immersion in agrobacteria suspension. In our case, such an approach has allowed to reduce the time needed for the production of transgenic plants from 18 to 10 months.

A virus-based expression system was also investigated as another way to shorten the production times. With the exception of a polerovirus (Miguel et al., 2016), no virus has been reported to infect carnivorous plants. The Tobacco Mosaic Virus (TMV) has been largely reported infect a broad range of plants. To check the virulence of TMV on *Nepenthes*, we made preliminary experiments using a wild-type strain of virus inoculated on leaves. Two weeks post-inoculation, although no symptoms appeared, the virus could be detected in the infected leaves but also on neighboring organs due to systemic propagation of the virus. These results led us to use TMV-based vectors for producing a recombinant GFP protein in *Nepenthes* (Gleba et al., 2005). The virus could not be produced in *Nepenthes* using an agroinfiltration approach and, therefore, the recombinant virus was propagated by infiltrating *N. benthamiana* leaves prior to inoculating *Nepenthes* leaves with a crude *N. benthamiana* leaf extract. This second strategy was successful, and we could recover recombinant GFP from TMV-infected *Nepenthes* leaves. This approach might now be improved by using other virus-derived vector such as a full length TMV-based vector, where the gene of interest is cloned between CP gene and MP gene and is controlled by the CP promotor (Lindbo, 2007).

Through this work, the proof of concept of a new plant system based on carnivorous plants for producing recombinant proteins is now established. The results obtained in this work now allow to evaluate a potential production of recombinant protein at larger scale for *Drosera*- and *Nepenthes*-based systems. In the case of *Drosera*, since 65 plants can be cultivated in one square meter, and digestive fluid can be harvested every 7–10 days, we assume that the production of GFP might reach on average 650 μg/m^2^/year. In *Nepenthes* case, plants present commonly about 15 mature pitchers. Assuming that 30 plants/m^2^ can be cultivated in suspension, 4.5 μg of GFP can be produced by one square meter every 2 weeks. Given that 26 collects can be done for 1 year, we can produce 117 μg of GFP /m^2^/year. Considering these small yields calculated per square meter, this platform is certainly not qualified for an industrial production scale but nonetheless demonstrates the potential interest of carnivorous plants for producing recombinant proteins. The magnitude of these estimated yields has been further confirmed with a human recombinant protein (Intrinsic Factor) for which the same experiments have been applied and have conducted to the obtention of similar yields. One prospect in the use of carnivorous plants for protein production might be to use *Nepenthes* endogen signal peptides that could increase the transcription of genes, the translation level of recombinant proteins and the secretion into the digestive fluid. Such small sequences formed by hydrophobic amino acids and localized at the N-terminal end of proteins have been documented (Faye et al., 2005) and the access to ‘omics’ databases might help identifying *Nepenthes* specific sequences. A Korean company has already patented the use of *N. alata* signal peptides to improve the secretion of recombinant proteins in trichomes of melon, cucumber, water melon, rape, and tobacco (Chang, 2005). Therefore, the interest of carnivorous plants for the production of recombinant proteins may lie in their outstanding organization of glandular tissues toward the excretion of endogenous proteins which could inspire biotechnologists for other plant-based platforms.

## Author Contributions

EN and FLB realized the molecular biology experiments and constructed the transgenic *Drosera*. SR and FLB performed the protein related experiments. SM did the transient expression experiments on *Nepenthes* plants. BM, EG, AH, and FRB supervised the research program. SM, FRB, and AH wrote the manuscript.

## Conflict of Interest Statement

The authors declare that the research was conducted in the absence of any commercial or financial relationships that could be construed as a potential conflict of interest.

## Footnotes

1 http://www.protalix.com

2 http://www.greenovation.com

3 http://www.araflora.com
