# Supplementary material for: Assessing Carnivorous Plants for the Production of Recombinant Proteins
Source: Front Plant Sci. 2019 Jun 19;10:793. doi: 10.3389/fpls.2019.00793 (PMC6593082; doi:10.3389/fpls.2019.00793)
Supplement: Supplementary file 1 [file Table_1.DOCX]

Supplementary Data:


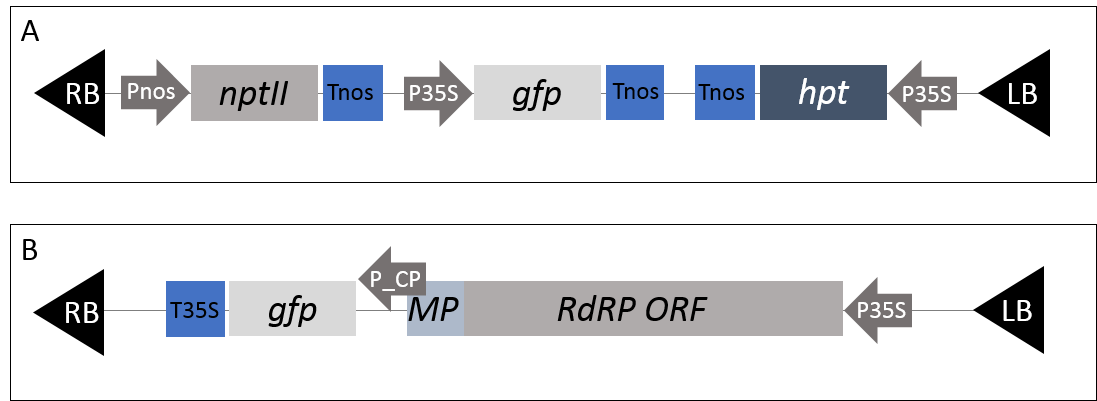


Figure 1: Representation of T-DNAs used in this study from pGWB2-*gfp* (A) and pMW388-*gfp* (B)

RB: right border; LB: left border; Tnos: terminator of nopaline synthase gene; Pnos: promotor of nopaline synthase gene; P35S: CaMV 35S promoter; *nptII:* neomycin phosphotransferase gene; *hpt*: hygromycin phosphotransferase; *gfp*: gene of green fluorescent protein with *A. thaliana* basic chitinase signal peptide indispensable to secretion of proteins ([U87974.1](https://www.ncbi.nlm.nih.gov/nucleotide/U87974.1?report=genbank&log$=nuclalign&blast_rank=1&RID=YB1HBER301R)) ; RdRP: TMV RNA-dependent RNA polymerase gene; MP: movement protein gene; P_CP: coat protein promoter; T35S: CaMV 35S terminator; Sequence accession of pGWB2 : AB289765.1; Sequence accession of pMW388 : JX971627.1
